# Modeling the Potential Distribution and Future Dynamics of Important Vector *Culex tritaeniorhynchus* Under Climate Change Scenarios in China

**DOI:** 10.3390/insects16040382

**Published:** 2025-04-03

**Authors:** Boyang Liu, Li Li, Zhulin Zhang, Haoyu Ran, Mingwei Xing

**Affiliations:** College of Wildlife and Protected Area, Northeast Forestry University, 26 Hexing Road, Harbin 150040, China; lilinefu@163.com (L.L.); 18944500626@163.com (Z.Z.); 15108363660@163.com (H.R.)

**Keywords:** *Culex tritaeniorhynchus*, vector-borne disease, climate change, ecological niche model

## Abstract

In the context of global warming, there is an increasing risk of the emergence and re-emergence of vector-borne diseases (VBDs). As one of the most important vectors, *Culex tritaeniorhynchus* can carry and transmit numerous human and animal infectious pathogens. To better understand the current distribution and possible future dynamics of *Cx. tritaeniorhynchus* in China, an ecological niche modeling approach (MaxEnt) was adopted to model its current and future habitat suitability. The most comprehensive dataset (1100 occurrence records) in China to date was established for model training. Multiple global climate models (GCMs) and climate change scenarios were introduced into the model to counter the uncertainties of future climate change. Based on the model prediction, *Cx. tritaeniorhynchus* currently exhibits high habitat suitability in southern, central, and coastal regions of China. It is projected that its suitable niche will experience continuous expansion, and the core distribution is anticipated to shift northward in the future 21st century (by the 2050s, 2070s and 2090s). Several environmental variables that reflect temperature, precipitation, and land-use conditions were considered to have a significant influence on the distribution of *Cx. tritaeniorhynchus*, among which annual mean temperature and urban land contribute the most to the model. Our study conducted a quantitative analysis of the shift and expansion of the future habitats of *Cx. tritaeniorhynchus*, providing references for vector monitoring and the prevention and control of VBDs.

## Simple Summary

*Culex tritaeniorhynchus* is an important vector insect of significant public health importance, capable of carrying and transmitting a series of infectious pathogens affecting humans and animals. Currently, it is mainly distributed in tropical and subtropical regions in China. As a climate-sensitive insect, it is speculated that the distribution of *Culex tritaeniorhynchus* will undergo significant changes in the context of global warming. In order to predict its current and future distribution within China, an ecological niche model was established based on occurrence records and environmental variables reflecting climate and land-use conditions. According to the modeling results, *Culex tritaeniorhynchus* is expected to establish new habitats in northern China in the 21st century. Our findings indicated that the ongoing global climate change will increase the risk of vector-borne diseases. Future efforts should prioritize the monitoring and control of disease vectors to prevent public health incidents.

## 1. Introduction

*Culex tritaeniorhynchus* Giles, 1901 is an important medical vector that transmits a variety of human and animal infectious disease pathogens. The most important zoonosis transmitted by *Cx. tritaeniorhynchus* is Japanese encephalitis (JE), which mainly causes infections in humans and pigs. Japanese encephalitis virus (JEV) is an important cause of viral encephalitis in Asia. JEV is believed to cause loss of more disability-adjusted life years than any other arthropod-borne virus, as it may lead to frequent neurological sequelae [1]. According to a systematic review of JE incidence, an estimated 67,900 JE cases may occur annually in JE-endemic countries, and approximately 50% of them were predicted to occur in China [2]. In the 1960s–1970s, several large-scale epidemics of JE occurred in China, with the highest incidence rate reaching 20.92/100,000 [3]. Since the vaccine was applied to the population in the 1980s, the nationwide pandemic has been brought under control. The early epidemic areas of JE in China were mainly located in the eastern coastal areas [4]. However, in recent years, the incidence rate of JE has been observed to rise in central and western China [5].

In addition to JE, there are various human or animal pathogens that can be transmitted by *Cx. tritaeniorhynchus*, such as Tembusu virus [6], Rift valley fever virus [7], Zika virus [8] and Lumpy skin disease virus [9]. Monitoring the distribution and pathogen-carrying status of this important vector is of great significance for disease prevention and control in the field of public health.

The ecological niche model (ENM) has been widely applied in predicting the potential distribution of species [10,11,12,13], especially those species (such as insects) that are significantly influenced by environmental factors, including meteorological conditions, landscape features, and other relevant aspects. ENMs can analyze the internal relationship between species’ adaptability to the environment based on the occurrence records of target species and environmental predictors with high resolutions [14]. Additionally, it can calculate and output the habitat suitability of species within the study area. Risk assessments of vector-borne diseases by modeling the distribution of arthropod vectors have been highlighted in a number of previous studies, such as *Culex* and *Aedes* mosquitoes for Rift valley fever [15], *Ixodes scapularis* for Lyme disease [16], and *Phlebotomus chinensis* for Leishmaniasis [17]. Modeling the distribution of vectors can help us discover their potential habitats and guide targeted monitoring efforts.

In the previous study [18], we predicted the distribution of *Cx. tritaeniorhynchus* in China by an ecological niche modeling approach based on 173 occurrence records (organized in 2018). As an extension of our previous study, we further improved the collection and collation of mosquito occurrence data and obtained the most comprehensive occurrence records of *Cx. tritaeniorhynchus* in China to date (1100 records with coordinates). Furthermore, land-use variables were introduced into the ENM and the future dynamics of potential habitat under climate change scenarios were modeled up to the end of this century. In recent years, an increasing number of studies have focused on the effect of future climate change on vector-borne diseases [19]. This study contributes to providing important references for the monitoring, prevention, and control of vector-borne infectious diseases such as JE. Additionally, it offers a perspective on future disease prevention and vector control strategies.

## 2. Materials and Methods

### 2.1. Cx. tritaeniorhynchus Occurrence Data

**Figure 1:**
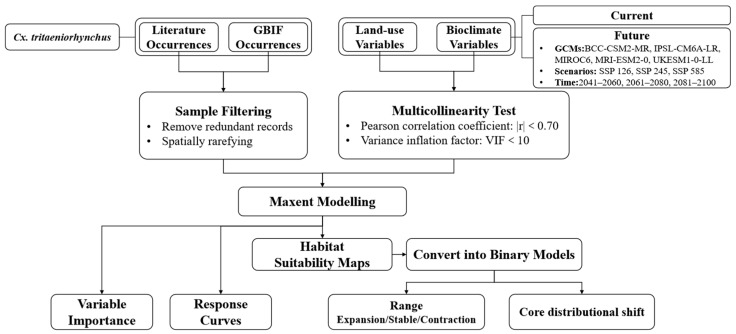
Flowchart of this study.

The flowchart of this study is shown in Figure 1.

**Figure 2:**
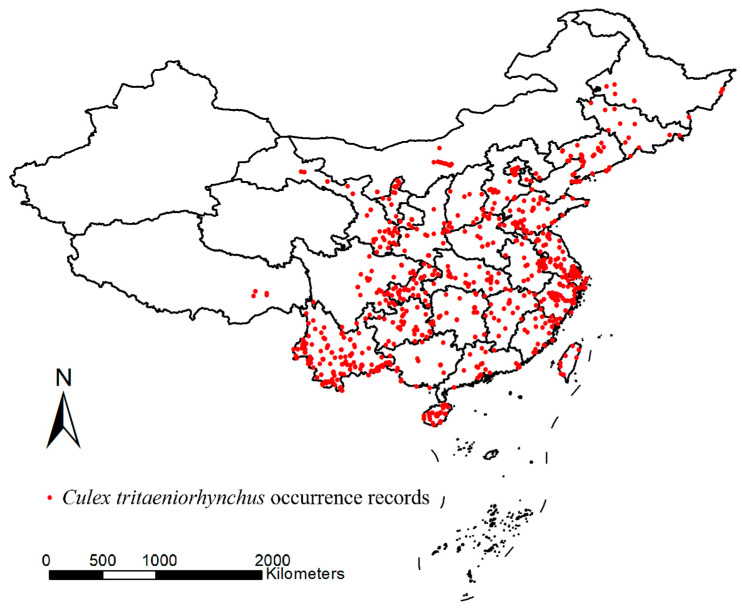
Occurrence records of *Cx. tritaeniorhynchus* in China (from 1957 to the present) (Details in Table S1). The map was created based on the base map GS(2024)0650 from the National Platform for Common Geospatial Information Services of China by using ArcGIS 10.2 (ESRI Inc., Redlands, CA, USA).

Occurrence data of *Cx. tritaeniorhynchus* in China were collected from two sources: (1) The literature retrieval. A comprehensive and systematic literature (both in English and Chinese) retrieval was performed on Web of Science, Scopus, ScienceDirect, PubMed and CNKI (Chinese National Knowledge Infrastructure). “*Culex tritaeniorhynchus* + collection/trap/occurrence/distribution/investigation/monitor/survey” were used as keywords for the retrieval. The time range of the obtained distribution records is from 1957 to the present. The literature containing only detailed information on distribution locations within China will be retained. If the literature contains the detailed coordinates of the mosquito sampling locations, they should be used directly. If the literature does not provide the coordinates but specifies the detailed sampling sites, Google Maps was used to conduct a search and determine the coordinates. A total of 913 occurrence records were obtained from 173 articles. (2) The Global Biodiversity Information Facility (GBIF) database. The GBIF database provides a public platform where occurrence records of any species can be uploaded and are available for open access and use by researchers. From this source, 187 occurrence records with coordinates within China were retrieved from the database. Finally, a database of the distribution of *Cx. tritaeniorhynchus* in China, consisting of 1100 occurrence records, was established (Figure 2).

Sampling points with the same coordinates at different dates, as well as occurrence records that are too close to each other, may both exist in the sample database. Data filtering was subsequently carried out by using the “Spatially rarefy occurrence data” module in SDM Toolbox v2.2 (a plugin for ArcGIS) [20]: (1) Remove spatially redundant occurrence records. In this step, 185 duplicates were removed. (2) Spatially rarefying occurrence records. Since the model would be conducted at the resolution of 2.5 arcmin (~5 km × 5 km), records in one grid cell were regarded as one record. To this end, 220 spatially autocorrelated points were removed.

### 2.2. Environmental Variables

Bioclimate variables and land-use variables were introduced as environmental predictive factors that have a significant impact on the distribution of mosquitoes (Table 1). Data for climate conditions were downloaded from WorldClim (https://www.worldclim.org, accessed on 5 January 2025). In this dataset, 19 bioclimate variables (Table 1) were provided to reflect the historical and future temperature and precipitation conditions. The future climate condition predictions provided by CMIP6 (Phase 6 of the Coupled Model Intercomparison Project) propose different levels of global climate change: Shared Socioeconomic Pathways (SSPs). The SSP1-2.6, SSP2-4.5, and SSP5-8.5 represent the low, moderate, and high emission scenarios, respectively, which were estimated to reach ~2.6, ~4.5, and ~8.5 W/m^2^ radiative forcing at stabilization in 2100 [21]. Global climate models (GCMs) developed by research institutions in various countries for assessing future climate change have been extensively used. Considering that the projection results of ENMs may vary depending on the GCMs used, a multi-model ensemble was used to reduce the uncertainties among GCMs [22]. Five GCMs (BCC-CSM2-MR, IPSL-CM6A-LR, MIROC6, MRI-ESM2-0 and UKESM1-0-LL) that are commonly used in meteorological modeling in China were selected in this study [23,24,25,26]. Both current and future variables have a high resolution of 2.5 arcmin (~ 5 km × 5 km).

Land-use variables with the same SSPs were obtained from the Land-Use Harmonization (LUH2) database (http://luh.umd.edu/index.shtml, accessed on 20 December 2024). The LUH2 dataset is also part of CMIP6. Projections of fractional global land-use patterns at 0.25° × 0.25° resolution up to 2100 were provided in this dataset. Twelve land-use variables were resampled using bilinear interpolation to the same resolution of bioclimate variables.

Multicollinearity usually occurs among the predictor variables in ENMs. In addition, reducing the number of variables has been proven to improve the accuracy and transferability of the model and mitigate the risk of overfitting [27,28]. In order to minimize the collinearity and retain valuable variables, a stepwise removal process was adopted by pre-running the model. All variables were incorporated into the pre-run model. (1) Pearson correlation coefficient is commonly used to detect the correlation among variables [29,30]. Correlated variables (|*r*| ≥ 0.70) with lower contribution in the pre-run model were considered to have poor predictive power and removed from the model. (2) After excluding all variables with significant correlations, further detection of potential multicollinearity was conducted based on the Variance Inflation Factor (VIF). Based on the same principle, variables with VIF ≥ 10 and lower contribution were eliminated until the VIF of the remaining variables were less than 10 [31]. VIF test was performed using the car package in R. The variables finally included in the model are marked as “Yes” in Table 1.

### 2.3. Maxent Modeling

Maxent 3.4.4 (https://biodiversityinformatics.amnh.org/open_source/maxent/, accessed on 20 December 2024) was applied to model the suitable habitats of *Cx. tritaeniorhynchus* in this study. It is a widely used model with superior predictive capabilities [32,33,34]. Sampling bias is a common problem in ENMs [35]. A commonly used approach to minimize the impact of sampling bias on modeling is generating background points (pseudo-absences) that have the same bias as the sampling points (presences) [35,36,37]. SDMtoolbox v2.4, a python-based tool for ArcGIS 10.2 (ESRI Inc.), was used to create Gaussian Kernel Density grids of sampling points to address the bias [20,38]. Background points were generated based on the bias grids, and 10,000 background points were recommended for Maxent modeling [32]. Several experimental runs with different spatial biases were carried out to determine the optimal modeling effect. In total, 25% percent of the occurrence points (randomly selected) were set as the test sample and 75% as the training sample. The other parameters remained the default settings of the Maxent program [39,40]. The average output of 10 replicates was taken as the final prediction for each model under each GCM. The receiver operating characteristic curve (ROC) and true skill statistic (TSS) were used to evaluate the model performance [41,42].

### 2.4. Suitable Habitat Shifts

The possible shift of suitable habitat of *Cx. tritaeniorhynchus* under future climate change scenarios was simulated through binary models, which were commonly used to convert continuous suitability maps into presence/absence maps. The threshold of “Maximum sensitivity plus specificity” was chosen to generate binary models which was considered as one of the best thresholds and provided accurate predictions [43,44,45]. The output pixels with predicted suitability greater than the threshold are regarded as “Suitable” and otherwise as “Unsuitable”. The differences between the future and current binary models were compared and marked as “Remain suitable” (Stable), “Become suitable” (Expansion), and “No longer suitable” (Contraction). Furthermore, the centroids of current and future predicted suitable ranges were calculated to explore the direction of core distributional shifts [46,47].

## 3. Results

### 3.1. Current Distribution of Cx. tritaeniorhynchus

**Figure 3:**
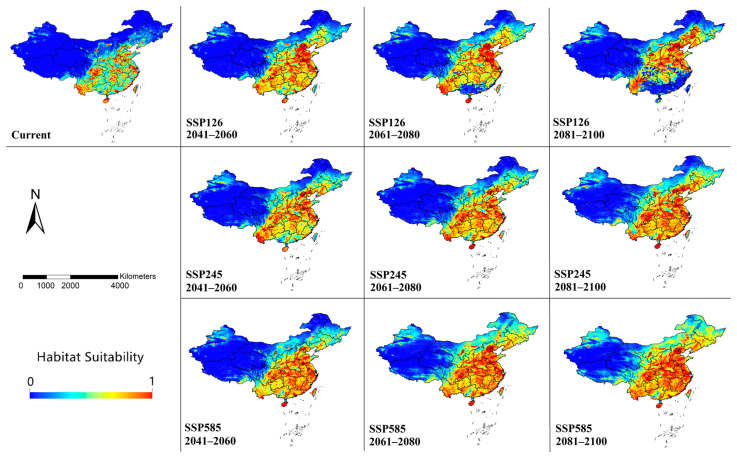
Modeled habitat suitability of *Cx. tritaeniorhynchus* in China by MaxEnt under climate change scenarios until the end of the 21st century. The maps were created using ArcGIS 10.2 (ESRI Inc.).

The modeling results of habitat suitability are shown in Figure 3. The current model performed well in representing the distribution of occurrence records. The AUC value of 10 replicates was 0.879 ± 0.017, and the TSS value was 0.650 ± 0.006, indicating that the model has good predictive performance.

Currently, *Cx. tritaeniorhynchus* is predicted to be widely distributed across China. High-suitability areas were modeled to be located mainly in southern (Hainan Island, southern Guangdong, eastern Guangxi and most of Yunnan), central (eastern Hunan and Hubei, Sichuan Basin, northern Henan, central Shaanxi and western Hebei), and coastal areas (Shandong, Jiangsu, Zhejiang, Fujian and Taiwan). Limited areas in the northeast (Liaoning, Jilin and Heilongjiang), north (Inner Mongolia) and west (Xinjiang and Tibet) were also predicted to have medium suitability for *Cx. tritaeniorhynchus*. It is noteworthy that, although there have never been reports of *Cx. tritaeniorhynchus* in Xinjiang, small parts of northern Xinjiang were predicted to have moderate habitat suitability.

### 3.2. Future Habitat Changes Based on Climate Change Scenarios

Under different climate change scenarios, the habitat suitability was predicted to change significantly with varying degrees until the end of the 21st century. In the SSP126 model, potential habitats with high suitability were modeled to show a significant trend of shifting northward. In the SSP245 and SSP 585 models, the changes in habitat suitability were similar, but with greater degrees of variation. In the currently low-suitable areas in the northeastern, northern, and western regions, a significant increase in suitability was predicted to occur in all SSPs. Even in the SSP585 model, almost the entire eastern part of China was projected to become suitable for *Cx. tritaeniorhynchus* by the end of the 21st century.

**Figure 4:**
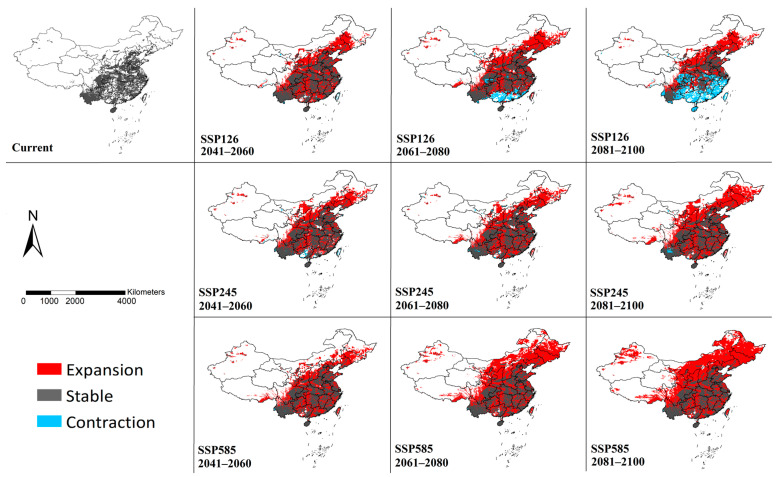
Modeled habitat changes of *Cx. tritaeniorhynchus* in China by MaxEnt under climate change scenarios. The maps were created using ArcGIS 10.2 (ESRI Inc.).

Binary models were generated based on the threshold of “Maximum sensitivity plus specificity”(Figure 4), which can more clearly reflect the details of suitable habitat changes in the future compared with the current. The suitable habitats were predicted to expand mainly towards the northern regions under the scenario of SSP126. Simultaneously, habitat contraction may occur in southern China. More suitable habitats will be established in central, northern and northeastern China under SSP245 and only limited areas in Yunan and Guangxi may experience a contraction of suitable habitats. In the prediction of the SSP585 model, not only will the vast majority of the eastern and northern regions become suitable habitats, but the suitable habitats will also expand westward. By the end of this century, large areas of habitats will also be established in northern Xinjiang and southeastern Tibet. And nearly no habitat contraction was detected under SSP585.

**Figure 5:**
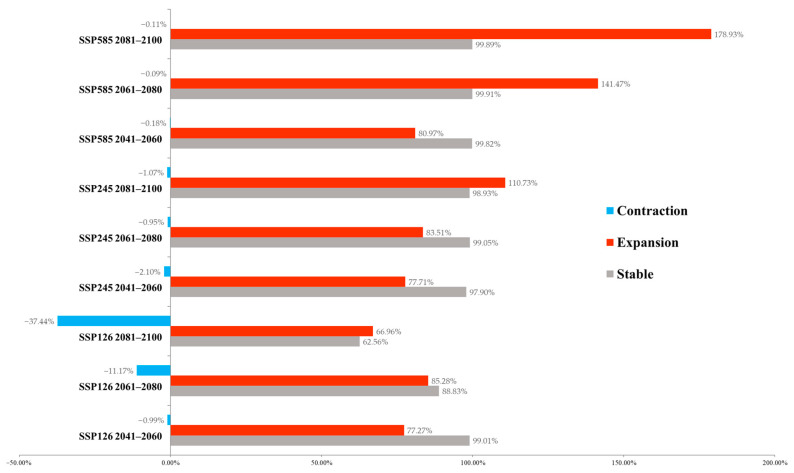
Estimated changes in modeled suitable habitat under climate change scenarios.

The change in suitable habitat area was calculated based on binary models (Figure 5). In the SSP126 model, with the passage of time, the area of the suitable habitat remaining stable will gradually decrease. Concomitantly, there will be a habitat contraction of up to 37.44% at the end of the 21st century. The most significant expansion was projected to occur in 2061–2080. In the SSP245 and SSP585 models, the vast majority of existing habitats will remain stable, and the expanded area will gradually increase. The most severe climate change scenario (SSP585) yields the highest predicted area of suitable habitats. Only very limited habitat loss was predicted to occur in SSP245 (0.95%~2.10%) and SSP585 (0.09%~0.18%).

### 3.3. Core Distributional Shifts

**Figure 6:**
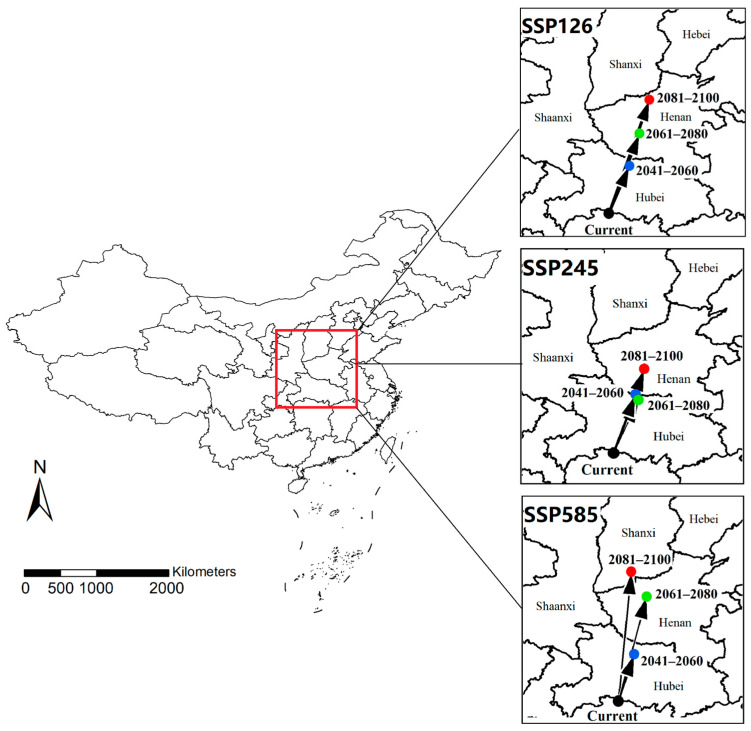
Modeled core distributional shifts under climate change scenarios. Blue/green/red dots represent the centroids of predicted suitable habitat in 2041–2060/2061–2080/2081–2100 under this scenario.

The trend of core distributional shifts was analyzed by calculating the centroids of modeled suitable habitats (Figure 6). In the SSP126 model, the direction of the core distributional shift was expected to remain consistent within the 21st century. The current centroid was located at the border of Hubei and Hunan provinces, in the central-southern region of China. It is predicted that it will continue to shift towards the northeast and reach northern Henan in 2081–2100. Although the shift direction is basically consistent with the SSP126 model, the SSP245 model provides the most conservative estimation. The modeled centroid in 2041–2060 and 2061–2080 are quite close, located at the border of Hubei and Henan provinces. Even by the end of the 21st century, the centroid will only shift to the central part of Henan. The direction and range of centroid shift differs under the SSP585 scenario. The modeled centroids in 2041–2060 and 2061–2080 shift towards the northeast, reaching northern Hubei and central Henan, respectively. However, in 2081–2100, the center is expected to shift northwestward (northward compared to its current position) into southern Shanxi.

### 3.4. Variable Importance

**Figure 7:**
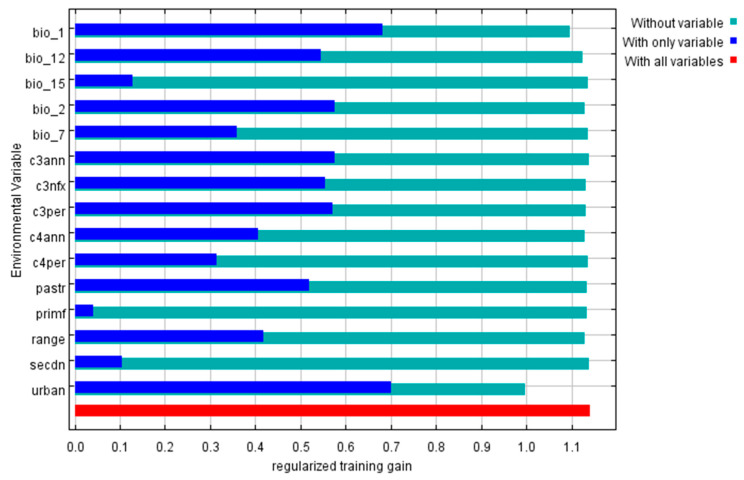
Result of the jackknife test. A longer blue bar or shorter green bar indicates greater relative importance to the model. Descriptions: bio_1: Annual mean temperature; bio_2: Mean diurnal range; bio_7: Temperature annual range; bio_12: Annual precipitation; bio_15: Precipitation seasonality; c3ann: C3 annual crops; c3nfx: C3 nitrogen-fixing crops; c3per: C3 perennial crops; c4ann: C4 annual crops; c4per: C4 perennial crops; pastr: Managed pasture; primf: Forested primary land; range: Rangeland; secdn: Potentially non-forested secondary land; urban: Urban land.

The jackknife test performed by the model demonstrates the importance of predictive variables (Figure 7). Variables with longer blue bars or shorter green bars are considered to have greater relative importance in the modeling. Among bioclimate variables, Bio 1 (Annual mean temperature), Bio 2 (Mean diurnal range) and Bio 12 (Annual precipitation) were considered to play important roles in impacting the distribution of *Cx. tritaeniorhynchus*. Land-use type of C3 crops (C3ann, C3nfx and C3per), pasture (pastr) and urban (urban) show great importance in the modeling.

## 4. Discussion

For the prevention and control of vector-borne diseases, the greatest challenge lies in understanding the distribution and population dynamics of vectors. According to the WHO guidance, the primary method for controlling most vector-borne diseases is to control the vectors directly through population-level interventions [48]. Periodic and systematic investigations of vectors enable the conduction of active surveillance of the potential pathogens carried by vectors. However, due to China’s vast territory, diverse climate types, and complex land-use conditions, it is extremely challenging to conduct comprehensive monitoring of insect vectors. Although China has established a vector-borne disease monitoring system since 2005, the system still has issues such as incomplete coverage areas, incomplete monitoring data, and insufficient communication and collaboration among departments. The implementation of ecological niche modeling research on insect vectors has the potential to provide guidance for more targeted monitoring efforts directed at vectors and pathogens carried by them.

The ongoing global warming, while giving rise to an increasing number of extreme climate events, is also subtly influencing the occurrence and distribution of vector-borne diseases. Climate change can influence the occurrence of vector-borne diseases through multiple direct or indirect pathways [49]. Higher temperatures are beneficial for shortening the reproduction cycle of mosquitoes [50,51], increasing mosquito bite rates [52] and shortening the extrinsic incubation period of pathogens carried by vectors [53], thereby posing a more severe risk of vector-borne disease to higher latitude regions in the foreseeable future. Increased precipitation was considered to provide more breeding sites for mosquitoes [51]. A warmer and more suitable climate prompts the migration of humans and other hosts to regions that were previously less suitable for survival. This also leads to changes in the distribution of mosquitoes. Studies in recent years have shown that significant changes have been monitored in the distribution of *Cx. tritaeniorhynchus* in northern China. For example, in 2018, the density of *Cx. tritaeniorhynchus* monitored in Beijing was more than 10 times the average of previous years [54]. And presence records of *Cx. tritaeniorhynchus* in high-altitude regions of Tibet were also reported in 2017 [55].

**Figure 8:**
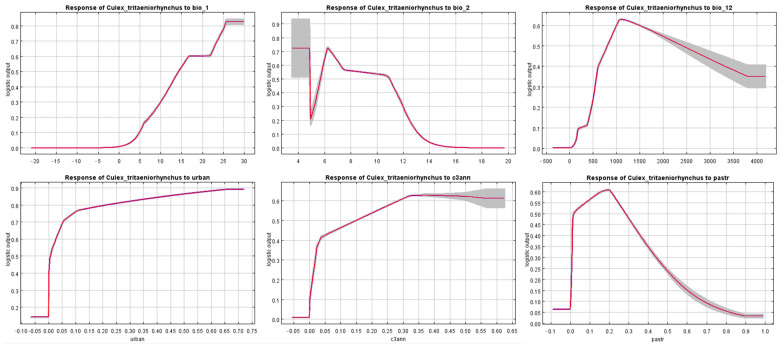
Response curves of variables with great importance. The red curves show the mean response of 10 replicates and the grey area represent +/− one standard deviation. Descriptions: bio_1: Annual mean temperature; bio_2: Mean diurnal range; bio_12: Annual precipitation; urban: Urban land; c3ann: C3 annual crops; pastr: Managed pasture.

According to the jackknife test, several variables with great importance were identified and their response curves (Figure 8) can reveal the inherent correlations between the distribution of *Cx. tritaeniorhynchus* and the variables. Bio 1 (Annual mean temperature) reflects its preference towards higher temperatures, which is also the reason why it is currently mainly distributed in the tropical and subtropical regions of China. Higher temperatures can accelerate its development process [56]. With the progression of global warming, the climatic conditions in the high-latitude regions of northern China will gradually become suitable for the survival of *Cx. tritaeniorhynchus*. The response curve of Bio 2 (Mean diurnal range) indicates that *Cx. tritaeniorhynchus* has difficulty adapting to large diurnal temperature differences, which is precisely the climatic characteristic of relatively cold low-latitude regions. Moderate to high annual precipitation (Bio 12) can bring about high habitat suitability. Water is necessary for the eggs of *Cx. tritaeniorhynchus* to maintain activity and hatch [57]. The demand for water in its life cycle was also reflected in the significant impact of croplands on its distribution. Firstly, crops serve as a sugar source for adult mosquitoes, providing essential sustenance for their survival and development [58]. Secondly, agricultural irrigation systems create aquatic environments that are conducive to mosquito oviposition and egg hatching [59]. For example, as the most popular staple in traditional Chinese agriculture, rice, with its extensive paddy fields, offers optimal conditions for mosquito reproduction. Urban land provides mosquitoes with an abundant supply of blood-feeding hosts (humans) as well as sufficient water sources and living shelters. Managed pastures with plants, livestock, and human residents also provide suitable conditions for the survival of mosquitoes. It should be noted that, apart from humans, pigs also serve as important blood-feeding hosts for *Cx. tritaeniorhynchus* [60]. With the development of the concepts of animal welfare and ecological farming [61], the breeding patterns of pasture pigs may pose a higher risk of vector-borne diseases in the future. Many variables among bioclimatic variables were excluded, while most land use variables were retained due to the absence of multicollinearity risk. This does not mean that certain variables that have been excluded have no impact on the distribution of mosquitoes. Instead, it is because the retained variables were considered to contain the vast majority of information from the excluded variables, and their contribution to the model has been proven to be greater in the pre-run.

As the primary vector for Japanese encephalitis (JE), clarifying the distribution of *Cx. tritaeniorhynchus* in China holds significant reference value for the early warning and monitoring of JE. The current habitat of *Cx. tritaeniorhynchus* predicted by our model is highly consistent with the spatial distribution of JE incidence data in China [4,62]. A small number of cases were reported in western and northern China. Most cases occur in the eastern and southern regions of China. Owing to the vaccination in endemic areas since the last century, the current incidence of JE in China has decreased significantly [63,64]. However, five provinces in southwest China were still considered highly epidemic areas for JE, including Henan, Chongqing, Sichuan, Guizhou, and Yunnan [62]. Our model indicates that these regions exhibit high suitability for *Cx. tritaeniorhynchus*. Moreover, Henan is worthy of special attention. Under different climate change scenarios, the centroid of the vector distribution was predicted to be within or near this province (Figure 5). Based on the model’s simulation results of the future suitable habitats for *Cx. tritaeniorhynchus*, it is evident that regions currently experiencing low-level or even no JE prevalence could potentially become suitable habitats for the vectors in the future. Although the extent of shift varies under different climate change scenarios, the overall direction of the centroid shift is towards northern regions. The rise in temperature makes the heat conditions in high-latitude regions more conducive to the growth and development of vectors. As a result, areas that were originally unsuitable may gradually become suitable for vectors. This indicates that the transmission risk of VBDs in the entire eastern half of China is likely to increase in the future. However, it is worth noting that in the projection for 2061–2100 under SSP126, habitats in southern China may experience contraction. This should be caused by the abnormal future changes in some variables under this scenario, which reminds us that we should not simply judge the future dynamics of insect vectors based on experience and model-based prediction is necessary.

Regions in western provinces not covered by the vaccination program of JE, such as Xinjiang, Tibet and Qinghai, suitable vector habitats were also predicted to emerge under severe climate change scenarios. Apart from JE, other VBDs also demand our attention. Lumpy skin disease was introduced into China in 2019 and then spread widely. At present, the distribution of its traditionally recognized transmission vectors (Stable flies, *Aedes aegypti*, etc.) in China is unclear; however, the evidence of the presence of LSDV in *Cx. tritaeniorhynchus* in China has been found in 2023 [9]. This provides us with new perspectives for exploring the transmission routes of LSDV in China. A similarly notable public health threat is the first isolation of the Zika virus from *Cx. tritaeniorhynchus* in China [8]. The spread of *Cx. tritaeniorhynchus* can lead to a noticeable increase in the exposure risk of VBDs. It is recommended that the above-mentioned regions strengthen vector surveillance and be prepared at all times to respond to emerging VBDs.

Compared with a previous study [65], this study constructs a dataset with a larger sample size (1100 occurrences in China vs. 139 occurrences in East Asia) by introducing a large number of the latest investigation data and domestic data in China. And the introduction and selection of predictive variables have been optimized, making the prediction for China more valuable as a reference. Moreover, future predictions based on climate change have also been performed in this study. Precisely forecasting future climate change and its consequential impacts on the distribution patterns of insect vectors is virtually an insurmountable task, attributable to the profusion of uncertainties. Nevertheless, modeling the current and future potential distributions of insect vectors based on different GCMs can still provide references for the prevention and control of VBDs. The samples in this study constitute the most complete and comprehensive occurrence database of *Cx. tritaeniorhynchus* within China to date. To take uncertainties into account as much as possible, we adopted the method of ensemble modeling. For the first time in modeling the future distribution of *Cx. tritaeniorhynchus* in China, bioclimate and land-use variables were introduced as environmental predictors based on multiple latest GCMs (BCC-CSM2-MR, IPSL-CM6A-LR, MIROC6, MRI-ESM2-0 and UKESM1-0-LL). Details of the predicted habitat changes and core distribution shifts were also analyzed. In addition to JE, the future monitoring and early warning of various human and animal pathogens transmitted by *Cx. tritaeniorhynchus* can also benefit from this study.

Our current study still has limitations. Although efforts have been made to counter the sampling bias, it is still impossible to completely avoid the potential impact of sampling bias in the modeling process. The modeled “Suitable habitats” were actually the ideal ecological niche for *Cx. tritaeniorhynchus*. The distribution of mosquitoes is influenced not only by the environmental factors considered in our model but also by many non-environmental factors, such as international travel and trade, interspecific competitions, vector control measures, insecticide resistance, etc. In addition to climate and land-use factors, there were several environmental factors that are also considered to be related to mosquito distribution, such as host population [66], topographic factors [67], and vegetation index [68]. It is indeed a pity that these influencing factors were not incorporated into our model. This is because our current study focused on the future vector distribution changes under climate change scenarios, and there is currently no corresponding future data available for those variables. Nevertheless, caution should be warranted for the long-term prediction results (especially in 2081–2100). This is because the future climate variables are themselves the results of simulations, along with the uncertainties associated with climate change. More valuable predictors will contribute to further improving the model and enhancing its prediction accuracy. Continuous surveillance of *Cx. tritaeniorhynchus* in the future is recommended in areas with high habitat suitability predicted by our model. The introduction of further monitoring data can be helpful for validating and improving the model.

## 5. Conclusions

Based on the most comprehensive dataset to date, we employed bioclimate and land-use variables as predictors to perform ecological niche modeling on the current and future potential habitat suitability of *Cx. tritaeniorhynchus* in China. The model was carried out based on multiple GCMs and fully took into account the uncertainties of future climate changes. Satisfactory modeling results were achieved for the current distribution of this important vector. In the 21st century, *Cx. tritaeniorhynchus* is assumed to expand its ecological niche under all scenarios. Northward shift and expansion of potential habitats is a common trend, with the SSP585 scenario yielding the most severe prediction. The results of our study can provide a reference for the monitoring and early warning of vector-borne diseases in the context of climate change, which will help policymakers to carry out targeted prevention and control efforts.

## Figures and Tables

**Table 1:** Environmental variables used in the model (Correlation matrix in Table S2).

Candidate Variables	Description	Source	Resolution	Included in the Final Model
Bio 1	Annual mean temperature	WorldClim	2.5 arcmin	Yes
Bio 2	Mean diurnal range			Yes
Bio 3	Isothermality (Bio 2/Bio 7)			
Bio 4	Temperature seasonality (Standard deviation*100)			
Bio 5	Maximum temperature of the warmest month			
Bio 6	Minimum temperature of the coldest month			
Bio 7	Temperature annual range (Bio 5-Bio 6)			Yes
Bio 8	Mean temperature of the wettest quarter			
Bio 9	Mean temperature of the driest quarter			
Bio 10	Mean temperature of the warmest quarter			
Bio 11	Mean temperature of the coldest quarter			
Bio 12	Annual precipitation			Yes
Bio 13	Precipitation of the wettest month			
Bio 14	Precipitation of the driest month			
Bio 15	Precipitation seasonality (Coefficient of variation)			Yes
Bio 16	Precipitation of the wettest quarter			
Bio 17	Precipitation of the driest quarter			
Bio 18	Precipitation of the warmest quarter			
Bio 19	Precipitation of the coldest quarter			
C3ann	C3 annual crops	LUH2	0.25 Degree	Yes
C3per	C3 perennial crops			Yes
C3nfx	C3 nitrogen-fixing crops			Yes
C4ann	C4 annual crops			Yes
C4per	C4 perennial crops			Yes
Primf	Forested primary land			Yes
Primn	Non-forested primary land			
Secdf	Potentially forested secondary land			
Secdn	Potentially non-forested secondary land			Yes
Pastr	Managed pasture			Yes
Range	Rangeland			Yes
Urban	Urban land			Yes

## Data Availability

The raw data supporting the conclusions of this article will be made available by the authors on request.

## Supplementary Materials

The following supporting information can be downloaded at: https://www.mdpi.com/article/10.3390/insects16040382/s1, Table S1: Sources of occurrence records; Table S2: Correlation matrix of variables.

## Abbreviations

The following abbreviations are used in this manuscript:

ENM	Ecological niche model
VBD	Vector-borne disease
SSP	Shared socioeconomic pathway
GCM	Global climate model
